# Elevation of plasma lysosphingomyelin-509 and urinary bile acid metabolite in Niemann-Pick disease type C-affected individuals

**DOI:** 10.1016/j.ymgmr.2018.03.005

**Published:** 2018-03-21

**Authors:** Ryuichi Mashima, Masamitsu Maekawa, Aya Narita, Torayuki Okuyama, Nariyasu Mano

**Affiliations:** a Department of Clinical Laboratory Medicine, National Center for Child Health and Development, 2-10-1 Okura, Setagaya-ku, Tokyo 157-8535, Japan; b Department of Pharmaceutical Sciences, Tohoku University Hospital, 1-1 Seiryo-machi, Aoba-ku, Sendai 980-8574, Japan; c Division of Child Neurology, Faculty of Medicine, Tottori University, 36-1 Nishi-cho, Yonago, Tottori 683-8504, Japan

## Abstract

Niemann-Pick disease type C (NPC) is a neurovisceral disorder associated with the accumulation of lipids such as cholesterol and sphingolipids. NPC is caused by either *NPC1* or *NPC2*, which encode lysosomal proteins located at membraneous and soluble fractions, respectively. For the past decade, the oxidation products of cholesterol, such as cholestane-3β,5α,6β-triol and 7-ketocholesterol, have been considered selective biomarkers for NPC. However, recent evidence has indicated numerous novel biomarkers for NPC, which raises the possibility that the diagnosis of NPC might be associated with the elevation of multiple lipid biomarkers, rather than a single biomarker. Sphingosylphosphorylcholine (SPC) has been suggested to be one such biomarker for NPC, in which elevated sphingomyelin is a potential precursor. Thus, we first performed a validation study of plasma SPC using LC-MS/MS. The results showed the following plasma concentrations in the NPC-affected and control individuals, respectively: 8.2 ± 2.8 nM (mean ± SD; median, 7.0 nM; max, 11.7 nM; min, 5.1 nM; *n* = 5) and 3.1 ± 1.4 nM (median, 2.9 nM; max, 4.8 nM; min, 1.5 nM; *n* = 7). We further extended the study to plasma lysophingomyelin-509 for NPC, a newly reported biomarker with uncharacterized chemical nature. Based on these result with plasma SPC as a surrogate marker, the value of mean of median of plasma lysophingomyelin-509 in NPC-affected individuals elevated at 65.2 (max, 73.2; min, 26.7; *n* = 5). Furthermore, the efficacy of plasma SPC and lysosphingomyelin-509 as promising biomarkers for this disorder was supported by the finding that the urinary concentration of 3β-sulfooxy-7β-*N*-acetylglucosaminyl-5-cholen-24-oic acid, an established biomarker for NPC, was also elevated in the NPC-affected individuals. These results suggest that a novel combination of plasma biomarkers, such as SPC and/or lysophingomyelin-509, and urinary bile acid metabolite could offer a promising platform for the diagnosis of NPC.

## 1 Introduction

Niemann-Pick disease type C (NPC) is a neurovisceral disorder caused by a defective mutation in either the *NPC1* (OMIM 607623) or the *NPC2* (OMIM 601015) gene [1,2]. The impaired egress of cholesterol from the late endosome/lysosomal compartment has been suggested to be a relevant mechanism for the pathogenesis of NPC. Early evidence showed consistently that NPC was associated with the accumulation of various lipids, including cholesterol in humans [3]. These lipids include a variety of cholesterol metabolites such as oxysterol, including cholestane-3β,5α,6β-triol and 7-ketocholesterol [[4], [5], [6], [7], [8], [9]], bile acids [[10], [11], [12], [13], [14]], and glucosylated cholesterol [15], respectively. The NPC1 protein is a membraneous protein in the lysosome, which facilitates the transportation of cholesterol from the lysosome to plasma membrane, whereas NPC2 is a soluble protein in the lysosome, which binds stoichiometrically to cholesterol. Based on these biochemical properties of NPC1 and NPC2, the mechanism(s) of NPC might be, at least partly, attributed to the failure of proper lipid trafficking in the cells [2]. This possibility was evidenced in several murine NPC models that showed positive therapeutic outcomes on the established NPC manifestations by treatment with cyclodextrin, a circular oligosaccharide that facilitates cholesterol transportation across the plasma membrane [[16], [17], [18]]. A recent study revealed the prevalence of classical NPC is 1/89,229, while the incidence of late-onset NPC incompletely predicted [19].

Both clinical and experimental evidence have indicated that the level of sphingomyelin is increased in NPC in the liver and spleen [2,3]. In mammals, the biosynthesis of sphingomyelin is initiated by serine and palmitoly-CoA in the endoplamic reticulum through the enzymatic action of serine: palmitoly-CoA transferase (EC 2.3.1.50) [20]. Sphingomyelin is a major sphingolipid that is located in the outer membrane of cells. Because sphingomyelin accumulates in NPC-affected individuals, the plasma concentration of lysosphingomyelin, also known as sphingosylphosphorylcholine (SPC), has been proposed as a biomarker for NPC [5,21,22]. An early study showed that the plasma concentration of SPC was correlated with that of cholestane-3β,5α,6β-triol and 7-ketocholesterol, both of which are widely accepted measures for the diagnosis of NPC [5]. A recent report documented that SPC was elevated in patients with acid sphingomyelinase deficiency as well as in patients with NPC [21]. Interestingly, this study also found that the selective accumulation of SPC in NPC was detectable only in the plasma, not in the dried blood spots [21]. Furthermore, lysosphingomyelin-509 is a novel biomarker with uncharacterized chemical nature, accumulating in the plasma of NPC-affected individuals [[21], [22], [23]]. Based on these results, we sought to determine whether the level of plasma SPC and lysosphingomyelin-509 might be correlated with another established biomarker for NPC. Among several candidate compounds, we focused on the recently discovered bile acid metabolite 3β-sulfooxy-7β-*N*-acetylglucosaminyl-5-cholen-24-oic acid (SNAG-Δ^5^-CA) because its elevation in NPC has been established [11].

## 2 Experimental procedure

### 2.1 Reagents

**Fig. 1:**
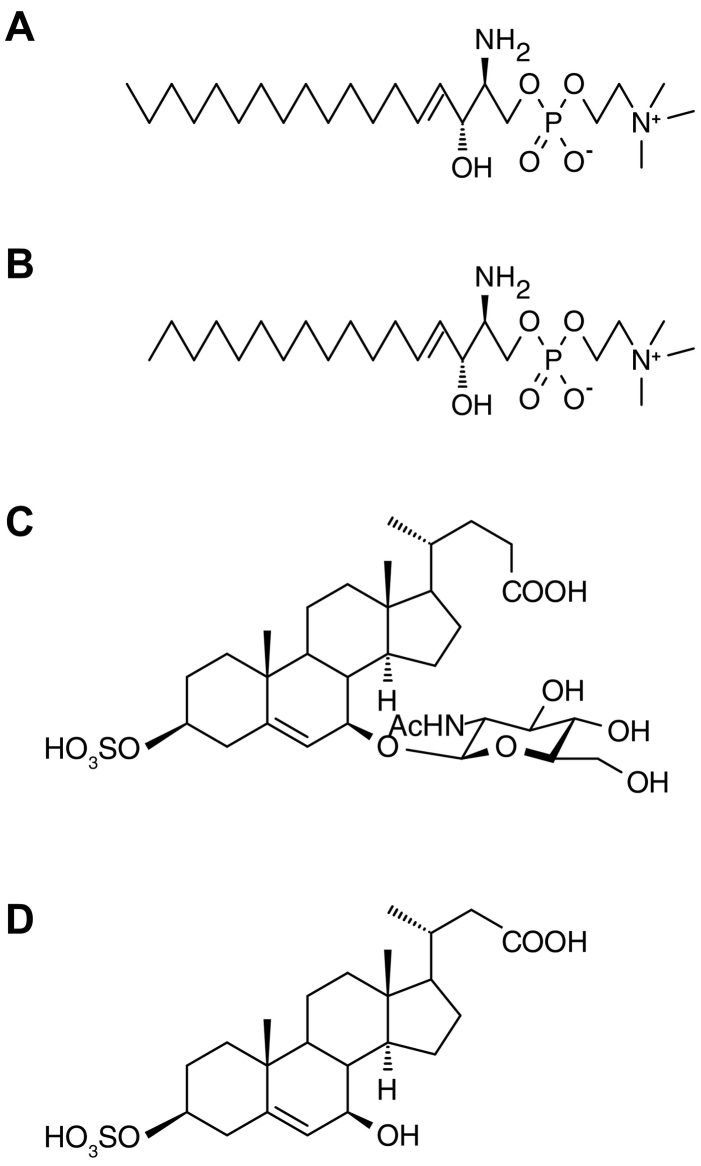
Chemical structures of compounds used in this study. (A) d-erythro-sphingosylphosphorylcholine (synthetic, SPC); (B) sphingosylphosphorylcholine (C17 base, IS_SPC_); (C) 3β-sulfooxy-7β-*N*-acetylglucosaminyl-5-cholen-24-oic acid (SNAG-Δ^5^-CA); (D) 3β-sulfooxy-7β-hydroxy-23-nor-5-cholenoic acid (IS_Bile acid_).

D-erythro-sphingosylphosphorylcholine (synthetic SPC) was purchased from Toronto Research Chemicals (Ontario, Canada). Sphingosylphosphorylcholine (C17 base, IS_SPC_) was purchased from Avanti Polar Lipids (Alabaster, Alabama, USA). Acetonitrile and methanol were purchased from Fischer Scientific (Tokyo, Japan). Deionized water was obtained from a Milli-Q water system (Millipore, Milford, MA, USA). Ammonium acetate and formic acid were purchased from Kanto Chemical (Tokyo, Japan). An Oasis HLB 96-well plate was purchased from Waters (Milford, MA, USA). 3β-Sulfooxy-7β-hydroxy-23-nor-5-cholenoic acid was synthesized and used as an internal standard for SNAG-Δ^5^-CA (IS_Bile acid_) as previously described [11,24]. The other reagents used in this study were of the highest grade commercially available. The chemical structures of the compounds used in this study are shown in Fig. 1.

### 2.2 Approval by institutional research ethics board

This study was approved by the Research Ethics Board of the National Center for Child Health and Development.

### 2.3 Patient specimens

All NPC-affected individuals were diagnosed by previously established method [11,14].

### 2.4 Solid-phase extraction

The preparation and analysis of plasma samples by LC-MS/MS assay with slight modifications was previously reported [5]. In brief, an aliquot of plasma (100 μL) was mixed with Solution 1 (900 μL, 75% water, 25% methanol, and 0.1% H_3_PO_4_), which contained 2.169 pmol IS_SPC_. This suspension then was loaded onto the Oasis HLB 96-well plate (30 μm, 30 mg, Waters), prewashed with hexane (1 mL) and methanol (1 mL), followed by equilibration with Solution 1 (1 mL × 2). After the sample was loaded, the column was washed with Solution 1 (1 mL) and Solution 2 (75% water and 25% methanol, 1 mL). Finally, the mixture of SPC and IS_SPC_ was eluted with Solution 3 (0.01% NH_3_ in methanol, 0.4 mL × 3). After the removal of solvent under N_2_ using a dry gas generator (Nihonseiki Co., Ltd., Osaka, Japan), the residue was reconstituted using Solution 4 (90% methanol, 10% water, and 0.1% formic acid, 60 μL).

### 2.5 Quantification of plasma SPC and lysosphingomyelin-509

An aliquot (1 μL) was injected onto a Nexara UPLC system linked to an LCMS8030plus mass spectrometer (Shimadzu, Kyoto, Japan). SPC, lysosphingomyelin-509 and IS_SPC_ were chromatographed on an InertSustainSwift C18 column (2.1 × 30 mm, 3 μm, GL Sciences, Tokyo, Japan) over 3 min using mobile phase A (5 mM ammonium acetate in methanol/water = 5/95) and mobile phase B (0.1% formic acid in methanol) at a flow rate of 0.5 mL/min. The temperature of the analytical column was maintained at 50 °C using a column oven CTO-10 (Shimadzu). The column was initially maintained using 60% mobile phase B for 0.5 min followed by a linear gradient using 60–100% mobile phase B at 0.5–1.5 min. Then the column was washed using 100% mobile phase B for 1.5–2.0 min followed by equilibration using 60% mobile phase B for 2.0–3.0 min. The data were acquired by multiple reaction monitoring (MRM) and the electrospray (ESI) positive mode. The chromatographic data were collected using software LabSolutions (Shimadzu). The instrumentation is described in detail in Supplementary Tables 1–3.

### 2.6 Quantification of a urinary bile acid metabolite SNAG-Δ^5^-CA

The concentration of SNAG-Δ^5^-CA was quantified as reported previously using an automated LC-MS/MS system with online-based sample purification [11]. In brief, an aliquot (50 μL) of a diluted urinary sample with the internal standard (100 μL of urine +100 μL of 2 μM IS_Bile acid_) was injected into a trapping column (Shim-pack MAYI-C8, 5 μm, 4.6 × 10 mm, Shimadzu, Kyoto, Japan) using binary mobile phases comprising aqueous 20 mM ammonium acetate (pH 5.5) and methanol (9/1, v/v) at a flow rate of 1.0 mL/min. After 3 min, SNAG-Δ^5^-CA was delivered to an YMC-Pack Pro C18 (5 μm, 2.0 × 150 mm, YMC, Kyoto) using 20 mM ammonium acetate (pH 5.5) and methanol (5/5, v/v) at a flow rate of 0.2 mL/min. The temperature of the columns was maintained at 40 °C.

An API 5000 mass spectrometer (AB Sciex, Framingham, MA) was used for the detection of SNAG-Δ^5^-CA. For selected reaction monitoring mode, we chose the combination of m/z 672.3 and m/z 97.0 for Q1 and Q3, respectively. The dwell time and collision energy were set at 250 ms and −70 V, respectively. The data were analyzed using the Analyst 1.4.1 software (AB Sciex). The instrumentation is described in detail in Supplementary Tables 4–6.

### 2.7 Statistical analysis

The data were expressed as mean ± SD. The mean values of the two groups were compared using a Student's *t*-test. The difference was considered statistically significant at *p* < 0.05.

## 3 Results

### 3.1 Assay validation

To quantify the plasma SPC and lysosphingomyelin-509 concentration with the highest possible sensitivity and precision, we selected SPC as the surrogate biomarker for lysosphingomyelin-509 using reversed-phase chromatography with MS/MS-based detection. Under our assay conditions, both SPC and IS_SPC_ showed linear responses ranging from 1 to 200 fmol (Supplementary Fig. 1). In this case, the intraday CV (%) was 7%, 6%, 6% for 216 nM, 108 nM, and 21.6 nM, respectively (*n* = 5). To examine the recovery of SPC in the biological samples, we first investigated the recovery of SPC in PBS as the blank matrix. Spiked SPC (11 nM) was detected in PBS with a recovery of 119% (Supplementary Fig. 2 and Table 1). Similarly, the recovery of spiked SPC (11 nM) in plasma was 120% under this assay condition. The interday CV (%) of the spiked SPC in plasma was 19% in 108 nM SPC, 6% in 11 nM, and 11% in 1 nM SPC (Table 2).

**Table 1:** Recovery and intraday assay precision of spiked SPC into PBS and plasma.

Matrix	Spiked SPC (nM)	Measured SPC (nM)	Recovery (%)	*n*	CV (%)
PBS	0	0.5	NA	5	10
PBS	1	1.6	101	5	12
PBS	11	13.3	119	5	15
PBS	108	100.1	93	5	14
Plasma	0	5.2	NA	5	7
Plasma	1	5.9	65	5	14
Plasma	11	18.1	120	5	14
Plasma	108	104.5	92	5	18

NA, not applicable.

**Table 2:** The interday CV (%) of plasma SPC using LC-MS/MS.

Matrix	Spiked SPC (nM)	Measured SPC (nM)			Mean (nM)	SD (nM)	*n*	CV (%)
		Run 1	Run 2	Run 3				
Plasma	0	5.2	4.0	5.7	5.0	0.9	3	18
Plasma	1	5.9	4.9	4.9	5.2	0.6	3	11
Plasma	11	18.1	17.7	16.0	17.3	1.1	3	6
Plasma	108	104.5	111.7	147.4	121.2	23.0	3	19

### 3.2 Plasma SPC and lysosphingomyelin-509 concentration in NPC-affected individuals

**Fig. 2:**
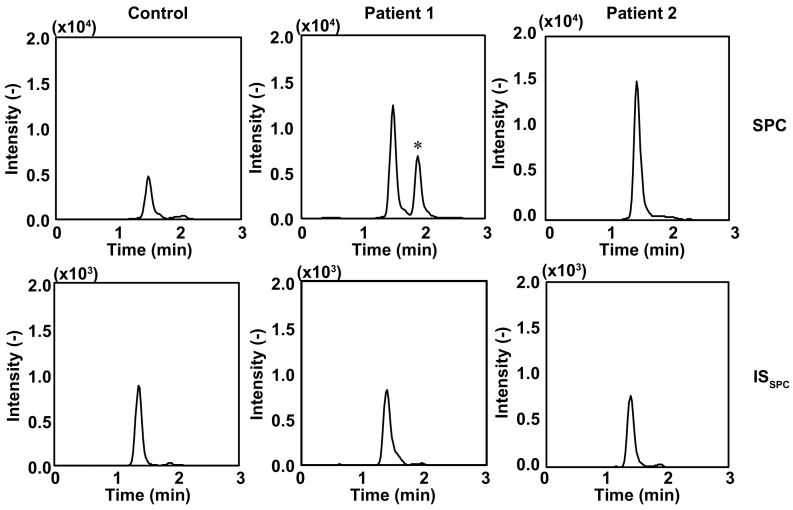
Representative chromatograms for SPC and IS_SPC_ from NPC-affected and control individuals. The concentration of SPC and IS_SPC_ was quantified using LC-MS/MS with MRM mode (SPC, 465.55 > 183.95; IS_SPC_, 451.4 > 183.90) as described in the experimental procedures. Both SPC and IS_SPC_ were detected as [M + H]^+^ ion using ESI positive mode. * denotes an uncharacterized peak.

**Fig. 3:**
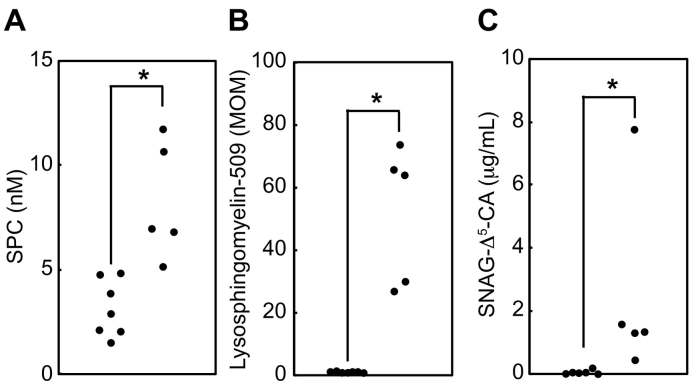
Elevation of plasma SPC and lysosphingomyelin-509 as well as urinary SNAG-Δ^5^-CA in NPC-affected individuals. (A) Concentrations of plasma SPC in NPC-affected individuals (*n* = 5) and controls (*n* = 7) were quantified using LC-MS/MS. (B) Concentrations of plasma lysosphingomyelin-509 in NPC-affected individuals (*n* = 5) and controls (*n* = 7) were quantified. (C) Concentrations of urinary SNAG-Δ^5^-CA in NPC-affected individuals (*n* = 5) and controls (*n* = 7) were quantified. *p < 0.05.

Based on the results of this validation study, we first examined the plasma SPC concentrations in the NPC-affected individuals. As shown in Fig. 2, the peak of SPC, which migrated at 1.5 min, was elevated in all NPC-affected individuals compared to the controls (Fig. 2, top), whereas the amount of IS_SPC_ remained unaltered (Fig. 2, bottom). In this study, the averaged plasma SPC concentrations in the NPC-affected individuals were 8.2 ± 2.8 nM (mean ± SD; median, 7.0 nM; max, 11.7 nM; min, 5.1 nM; *n* = 5), whereas in the controls, the averaged plasma SPC concentrations were 3.1 ± 1.4 nM (median, 2.9 nM; max, 4.8 nM; min, 1.5 nM; *n* = 7) (Fig. 3A). Thus, the increase in the averaged SPC concentration in the NPC-affected individuals was 2.6-fold higher than in the controls (Table 3). Similarly, the median value of multiple of median of plasma lysosphingomyelin-509 in NPC-affected individuals was 65.2-fold (max, 73.2; min, 26.7; *n* = 5) (Fig. 3B). To further ensure that the accumulation of these two biomarkers were consistent with previously characterized biomarker for NPC, we sought to determine whether SNAG-Δ^5^-CA, an established biomarker for NPC in urine, might be elevated in NPC-affected individuals. Thus, we quantified urinary SNAG-Δ^5^-CA concentrations in samples taken from the same individuals using 3β-sulfooxy-7β-hydroxy-23-nor-5-cholenoic acid as IS_Bile acid_. The LC-MS/MS assay revealed that the SNAG-Δ^5^-CA concentrations in the NPC-affected samples were 2477 ± 2968 ng/mL (median, 1332 ng/mL; max, 7731 ng/mL; min, 449 ng/mL; *n* = 5), whereas those in the control samples were 39 ± 73 ng/mL (mean ± SD; median, 18 ng/mL; max, 204 ng/mL; min, 0 ng/mL; *n* = 7) (Fig. 3C).

**Table 3:** Reported concentrations of plasma SPC in NPC-affected individuals.

Investigator	Plasma SPC (nM)	Cutoff (nM)	Increase (fold)	*n*	Extraction	Country/area	Ref
Welford RW et al.	7.23–69.73	Not determined	2.8	57	SPE	Europe and Brazil	[5]
Kucher L et al.	16–111	Not determined	3.3	15	Butanol	Not described	[21]
Polo G et al.	Approx 20–80	16.8	3.4	11	N/A	Italy	[22]
Mashima R et al.	5.1–11.7	Not determined	2.6	7	SPE	Japan	This study

SPE, solid-phase extraction.

### 3.3 The correlation between SPC and lysosphingomyelin-509 and a bile acid metabolite SNAG-Δ^5^-CA

**Fig. 4:**
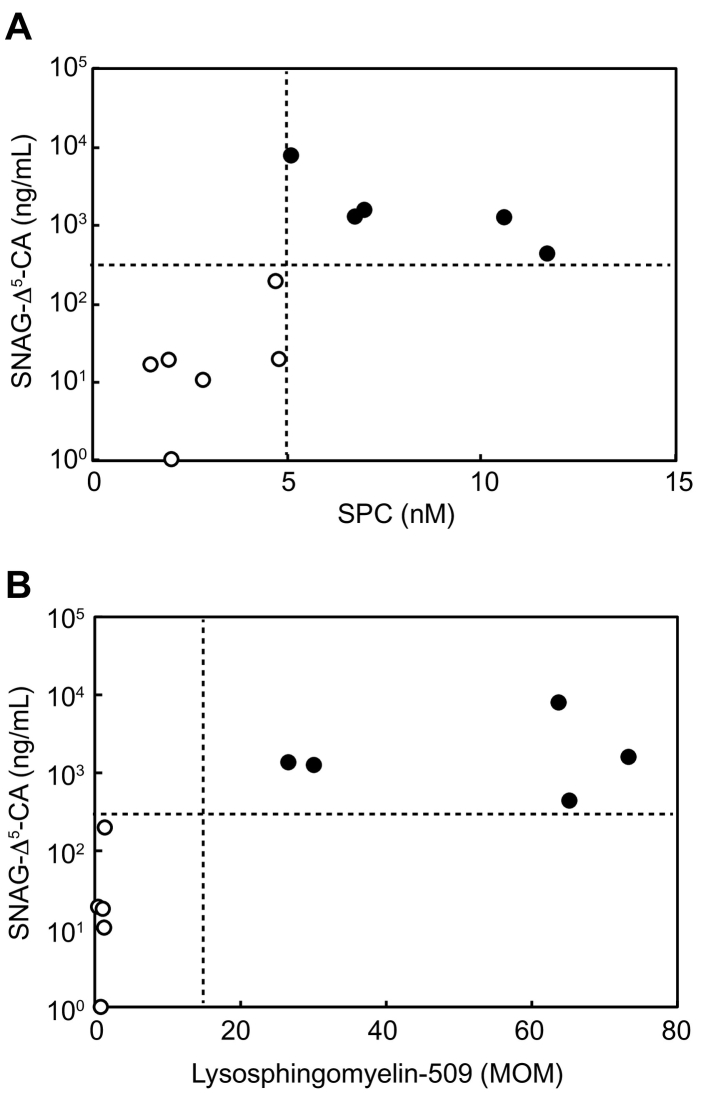
Correlation between the concentrations of biomarkers in plasma and in urine in NPC-affected individuals. (A) The plasma SPC concentration and urinary SNAG-Δ^5^-CA concentration was presented. (B) The plasma lysosphingomyelin-509 concentration and urinary SNAG-Δ^5^-CA concentration was presented. The SNAG-Δ^5^-CA concentration was quantified using 3β-sulfooxy-7β-hydroxy-23-nor-5-cholenoic acid as an internal standard according to Maekawa et al. [11].

Fig. 4A shows a summary of the correlation between plasma SPC and urinary bile acid metabolite SNAG-Δ^5^-CA. As shown, all NPC-affected individuals examined in this study exhibited higher concentrations of both SPC and SNAG-Δ^5^-CA. These results demonstrated that the combined quantification of plasma SPC and urinary bile acid metabolite SNAG-Δ^5^-CA using LC-MS/MS provided a more solid diagnostic basis for NPC than the single biomarkers did. Similarly, the correlation of plasma lysosphingomyelin-509 and urinary SNAG-Δ^5^-CA concentrations was clearly demonstrated (Fig. 4B). Within our examination, the sensitivity and specificity of plasma biomarkers, such as SPC and lysosphingomyelin-509, and of urine metabolites in NPC was 1 and 1, respectively.

## 4 Discussion

NPC is a lysosomal storage disorder caused by the pathogenic deficiency of either the *NPC1* or *NPC2* gene. Although the precise mechanism of the occurrence and development of NPC induced by these two genes remains uncertain, the accumulation of cholesterol in late endosome/lysosome has been suggested to be the precondition of this disorder. The hallmark of NPC involves the accumulation of cholesterol and its oxidation products. In support, previous studies showed that the elevation of plasma oxysterols, such as cholestane-3β,5α,6β-triol and 7-ketocholesterol, were accepted as the diagnostic measure of NPC. In fact, the specificity of plasma cholestane-3β,5α,6β-triol and 7-ketocholesterol for NPC were 1.0 and 0.9984, respectively [25]. The overall correlation between previously proposed biomarkers for NPC was summarized in Table 4.

**Table 4:** Correlation between several biomarkers in NPC-affected individuals.

Investigator	Year	Biomarkers	Correlation	Ref
Porter FD et al.	2010	Plasma cholestane-3β,5α,6β-triol vs plasma 7-ketocholesterol	Yes	[25]
Welford RW et al.	2014	Plasma SPC vs plasma cholestane-3β,5α,6β-triol	Yes	[5]
	2014	Plasma SPC vs plasma glucosylsphingosine	No	[5]
Jiang X et al.	2016	Plasma bile acid A vs plasma bile acid B	Yes	[13]
	2016	Plasma cholestane-3β,5α,6β-triol vs DBS bile acid B	Yes	[13]
Mashima R et al.	2018	Plasma SPC vs urinary bile acid metabolite SNAG-Δ^5^-CA	Yes	This study
	2018	Plasma lysosphingomyelin-509 vs urinary bile acid metabolite SNAG-Δ^5^-CA	Yes	This study

Bile acid A, 5α-cholanic acid-3β,5α,6β-triol; bile acid B, 5α-cholanic acid-3β,5α,6β-triol *N*-(carboxymethyl)-amide.

Bile acid metabolites, which are a series of compounds that originate in bile acids, have received much attention as biomarkers for NPC [[10], [11], [12], [13], [14],^26^,^27^]. A milestone study was performed by Alvelius et al., which showed that bile acid metabolites in NPC-affected individuals were elevated by GC–MS [10]. Most bile acids produced in the body are excreted, whereas a small portion is known to circulate in the blood [28]. A recent study reported the correlation between cholestane-3β,5α,6β-triol in the plasma and the bile acid metabolite 5α-cholanic acid-3β,5α,6β-triol *N*-(carboxymethyl)-amide in a dried blood spot, which suggested the potential of circulating bile acid metabolites to be a novel biomarker for NPC [13]. The present study demonstrated a positive correlation between plasma biomarkers, such as SPC and/or lysosphingomyelin-509, and the urinary bile acid metabolite SNAG-Δ^5^-CA, which suggests that separate biological specimens (i.e., the plasma and urine) from one individual allowed for double-checking the results of diagnosis using a biochemical platform. It is generally anticipated that the prevalence of NPC is lower than other lysosomal-storage diseases such as Pompe disease and Fabry disease. This could be inversely correlated with the number of previously identified pathogenic mutations of *NPC1* and *NPC2*, thus, so far the database of pathogenic mutations in *NPC1* and *NPC2* may not contain all pathogenic data. Thus, the combined assay of plasma biomarkers, such as SPC and/or lysosphingomyelin-509, and urinary bile acid metabolite SNAG-Δ^5^-CA may provide a much solid diagnostic basis for the pathogenicity of NPC, especially when previously uncharacterized genomic mutation(s) are involved.

Although the mechanism of SPC formation in mammals still requires full elucidation, at least three distinct pathways have been postulated [29]. First, sphingomyelin deacylase (EC 3.5.1.109) catalyzes the formation of SPC from sphingomyelin in the cis-Golgi apparatus [30]. Second, the synthesized SPC translocates across the plasma membrane followed by conversion to sphingosine-1-phosphate by phospholipase D activity through nucleotide pyrophosphatase/phosphodiesterases [31]. Third, this extracellular SPC also undergoes sphingosine-1-phosphate through autotaxin [32]. Based on these potential mechanisms, it is anticipated that the accumulation of SPC at least in part might be linked to sphingomyelin, which is a well-established biomarker for NPC [2]. In fact, to date, only one study performed by Welford et al. has demonstrated that plasma SPC concentration was correlated with plasma cholestane-3β,5α,6β-triol but not with glucosylsphingosine in NPC-affected individuals (Table 4) [5]. At this stage, while the mechanism of biotransformation of lysosphingomyelin-509 has attracted attention due to the higher specificity of diagnosis for NPC, the details of this awaits further investigation.

In summary, the present study provided evidence that plasma SPC was elevated in NPC-affected individuals using LC-MS/MS. More strikingly, plasma lysosphingomyelin-509 concentration in the NPC-affected individuals was strongly increased in NPC-affected individuals. This concentration was correlated with the urinary bile acid metabolite SNAG-Δ^5^-CA, thus, a combination of biomarkers from two separate biological sources, such as plasma and urine may provide a more solid biochemical basis for the diagnosis of NPC.

## Appendix A Supplementary data

Supplementary Tables Image 1

Supplementary Figures Image 2
