# Supplementary material for: Elevation of plasma lysosphingomyelin-509 and urinary bile acid metabolite in Niemann-Pick disease type C-affected individuals
Source: Mol Genet Metab Rep. 2018 Mar 21;15:90–5. doi: 10.1016/j.ymgmr.2018.03.005 (PMC6047109; doi:10.1016/j.ymgmr.2018.03.005)
Supplement: Supplementary file 1 — Supplementary Tables [file mmc1.docx]

**Supplementary Table 1**

Instrument parameters for chromatograph for SPC quantification in human plasma by reversed-phase chromatography using LC-MS/MS.

| LC | UPLC chromatograph Nexara (Shimadzu, Kyoto, Japan) |
| --- | --- |
| Column | InertSustainSwift C18 (GL Scicences, Tokyo, Japan) |
|  | Particle diameter: 2.1 μm |
|  | Internal diameter: 3 mm |
|  | Length: 30 mm |
| Column temperature | 50°C |
| Wash solvent | Methanol |
| Mobile phase A | 5 mM Ammonium acetate in methanol/water (5/95) |
| Mobile phase B | 0.1% formic acid in methanol |
| Gradient (%B) | 0-0.5 min: 60% B |
|  | 0.5-1.5 min: 60-100% B |
|  | 1.5-2.0 min: 100% B |
|  | 2.01-3.0 min: 60% B |
| Flow rate | 0.5 mL/min |
| Injection volume | 1 μL |
| Injection mode | Direct injection |
| Autosampler temperature | 4°C |

**Supplementary Table 2**

Instrument parameters for mass spectrometer for SPC quantification in human plasma by reversed-phase chromatography using LC-MS/MS.

| MS | LCMS8030plus (Shimadzu, Japan) |
| --- | --- |
| Interface | ESI |
| Polarity | Positive |
| Capillary voltage | 4.5 kV |
| Heatblock temperature | 400°C |
| Desolvation temperature | 250°C |
| Flow rate of nebulizer gas | 3.0 L/min |
| Flow rate of drying gas | 15 L/min |
| Analyzing mode | MRM |
| Data format | Centroid |

**Supplementary Table 3**

Product ion, precursor ion, cone voltage and collision energy for SPC quantification in human plasma by reversed-phase chromatography using LC-MS/MS.

|  | Precursor ion  (*m/z*) | Product ion  (*m/z*) | Dwell time  (ms) | Cone  (V) | CE  (V) | Q3  (V) |
| --- | --- | --- | --- | --- | --- | --- |
| Sphingosylphosphorylcholine (SPC) | 465.55 | 183.95 | 100 | -20 | -25 | -20 |
| Lysosphingomyelin-509 | 509.5 | 183.95 | 100 | -20 | -25 | -20 |
| Sphingosylphosphorylcholine (C17 base, IS_SPC_) | 451.4 | 183.90 | 100 | -12 | -24 | -18 |

**Supplementary Table 4**

Instrument parameters for chromatograph for SNAG-Δ^5^-CA quantification in human urine by reversed-phase chromatography using LC-MS/MS.

| LC | Nanospace SI-2 (Shiseido, Tokyo, Japan) |
| --- | --- |
|  |  |
| Trapping column | Shim-pack MAYI-C8 (Shimadzu, Kyoto, Japan) |
|  | Particle diameter: 5 μm |
|  | Internal diameter: 4.6 mm |
|  | Length: 10 mm |
| Mobile phase A | 20 mM Ammonium acetate (pH 5.5) |
| Mobile phase B | Methanol |
| Composition | 90% B |
| Flow rate | 1.0 mL/min |
|  |  |
| Analytical column | YMC-Pack Pro C18 (YMC, Tokyo, Japan) |
|  | Particle diameter: 5 μm |
|  | Internal diameter: 2.0 mm |
|  | Length: 150 mm |
| Column temperature | 40 °C |
| Mobile phase A | 20 mM Ammonium acetate (pH 5.5) |
| Mobile phase B | Methanol |
| Composition | 50% B |
| Flow rate | 0.2 mL/min |

**Supplementary Table 5**

Instrument parameters for mass spectrometer for bile acid metabolite SNAG-Δ^5^-CA quantification in human urine by reversed-phase chromatography using LC-MS/MS.

| MS | API 5000 (AB Sciex, Framingham, MA) |
| --- | --- |
| Interface | ESI |
| Polarity | Positive |
| Ion spray voltage | -4500 V |
| Declustering potential | -80 V |
| Turbospray gas temperature | 700°C |
| Desolvation temperature | 250°C |
| Gas | Nitrogen |
| Flow rate of curtain gas | 25 units |
| Flow rate of gas 1 | 40 units |
| Flow rate of gas 2 | 60 units |
| Flow rate of collision gas | 6 units |
| Analyzing mode | SRM |
| Data format | Centroid |

**Supplementary Table 6**

Product ion, precursor ion, cone voltage and collision energy for SNAG-Δ^5^-CA quantification in human urine by reversed-phase chromatography using LC-MS/MS.

|  | Precursor ion  (*m/z*) | Product ion  (*m/z*) | Dwell time  (ms) | Collision energy  (V) |
| --- | --- | --- | --- | --- |
| SNAG-Δ^5^-CA | 672.3 | 97.0 | 250 | -70 |
| 3β-Sulfooxy-7β-hydroxy-23-nor-5-cholenoic acid (IS_Bile acid_) | 455.6 | 97.0 | 250 | -70 |

SNAG-Δ^5^-CA, 3β-sulfooxy-7β-*N*-acetylglucosaminyl-5-cholen-24-oic acid.
