# Supplementary figures and images for: Elevation of plasma lysosphingomyelin-509 and urinary bile acid metabolite in Niemann-Pick disease type C-affected individuals
Source: Mol Genet Metab Rep. 2018 Mar 21;15:90–5. doi: 10.1016/j.ymgmr.2018.03.005 (PMC6047109; doi:10.1016/j.ymgmr.2018.03.005)

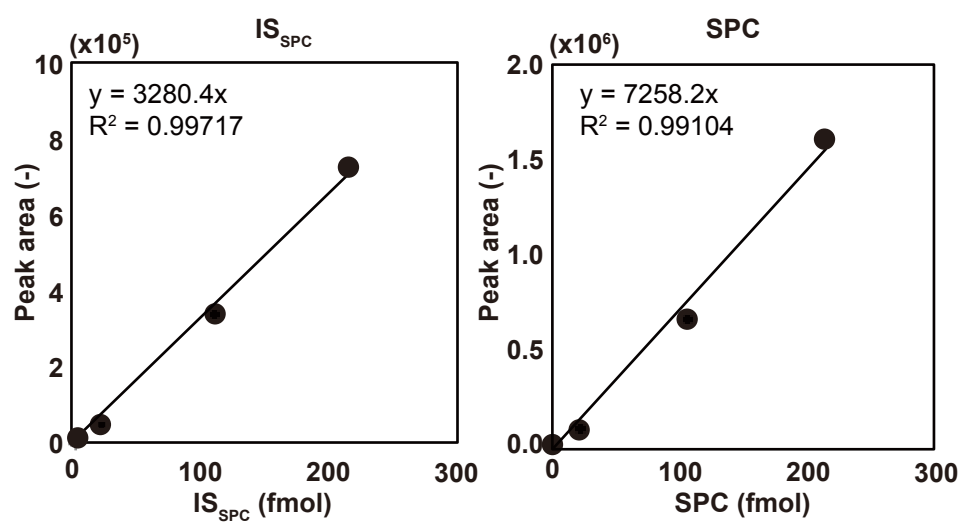

Supplementary Fig. 1  
Mashima R et al

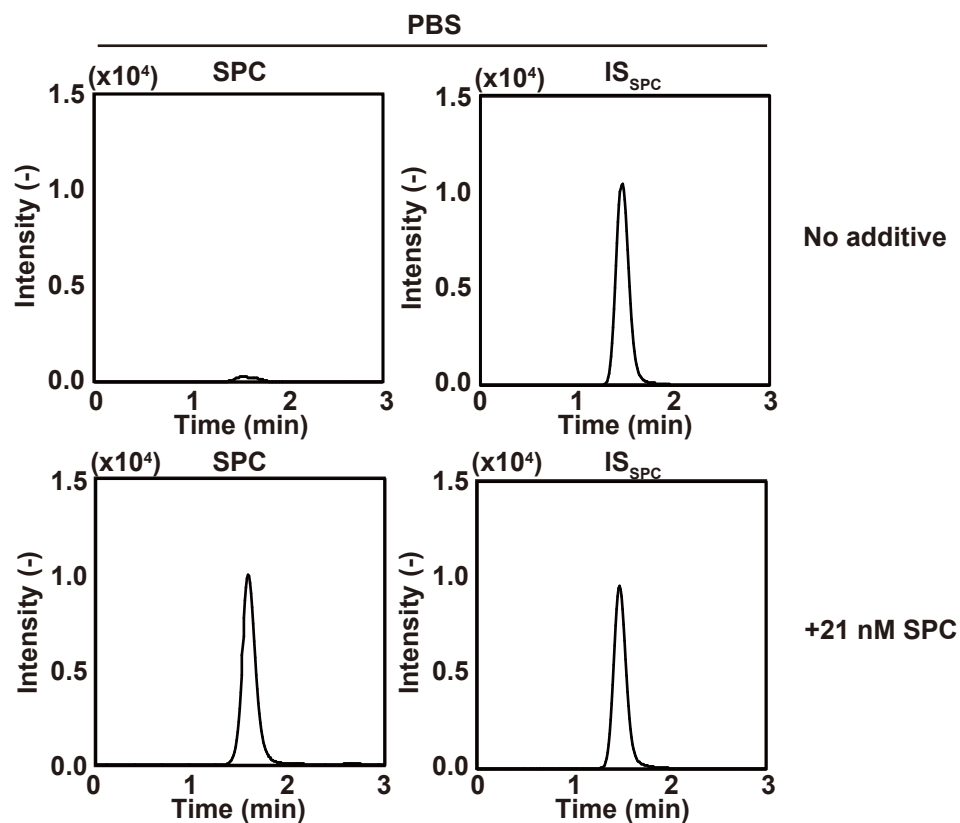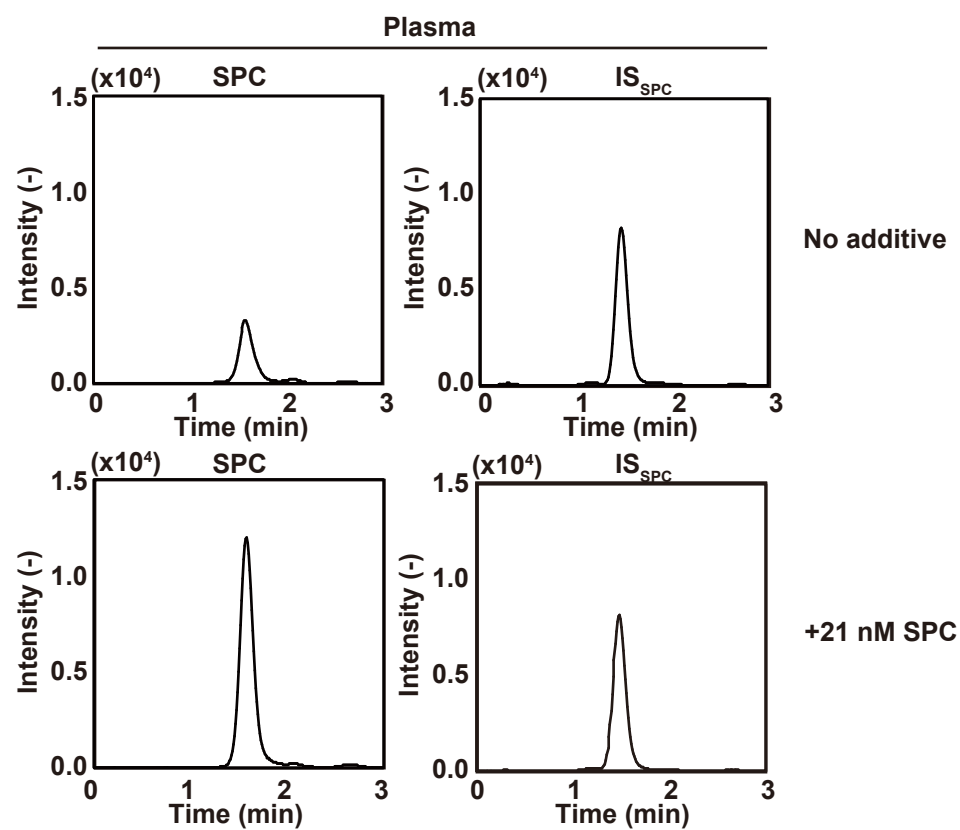

Supplement: Supplementary file 2 — Supplementary Figures [file mmc2.pdf]
